# The Role of Adipokines in Tumor Progression and Its Association with Obesity

**DOI:** 10.3390/biomedicines12010097

**Published:** 2024-01-03

**Authors:** Jae Won Kim, Jun Hyeok Kim, Yoon Jae Lee

**Affiliations:** Department of Plastic and Reconstructive Surgery, Yeouido St. Mary’s Hospital, College of Medicine, The Catholic University of Korea, Seoul 07345, Republic of Korea; yoamelia88@gmail.com (J.W.K.); hyeoggy@gmail.com (J.H.K.)

**Keywords:** adipokine, obesity, tumorigenesis

## Abstract

Obesity is a well-established risk factor for various malignancies and emerging evidence suggests that adipokines play a pivotal role in linking excess adiposity to tumorigenesis. Adipokines are bioactive molecules secreted by adipose tissue and their altered expression in obesity contributes to a pro-inflammatory, pro-angiogenic, and growth-promoting microenvironment conducive to tumorigenesis. Leptin, a key adipokine, activates survival and proliferative signaling pathways whereas adiponectin exhibits tumor-suppressive effects by inducing apoptosis and cell cycle arrest. Visfatin has also been documented to promote tumor growth, angiogenesis, migration, and invasion. Moreover, emerging studies suggest that adipokines, such as resistin, apelin, and chemerin, which are overexpressed in obesity, may also possess oncogenic functions. Despite advancements in our understanding of the roles of individual adipokines in cancer, the intricate interplay and crosstalk between adipokines, tumor cells, and the tumor microenvironment remain complex and multifaceted. This review highlights the evolving knowledge of how adipokines contribute to obesity-related tumorigenesis, shedding light on the potential of targeting adipokine signaling pathways as a novel therapeutic approach for obesity-associated cancers. Further research on the specific mechanisms and interactions between adipokines and tumor cells is crucial for a comprehensive understanding of obesity-associated cancer pathogenesis.

## 1. Introduction

Once regarded as an energy storage depot, adipose tissue underwent a paradigm shift in understanding and is now recognized as an active endocrine organ. They possess a significant capability to secrete a diverse array of bioactive molecules collectively referred to as adipokines [1]. Adipokines, a group of cytokines and bioactive mediators primarily secreted by adipocytes, play pivotal roles in regulating glucose and energy homeostasis, inflammation, and immune responses [2]. Notably, the influence of adipokines extends beyond their local activities within the adipose tissue microenvironment, as they also exert systemic effects on distant organs and tissues [3]. Owing to their pleiotropic effects, adipokines have emerged as crucial players in the intricate interplay between adipose tissue and other physiological systems and affect various pathophysiological processes, including tumorigenesis. A critical domain in which adipokines are gaining prominence is their involvement in tumorigenesis, where their pleiotropic effects are instrumental in shaping the landscape of cancer development.

Obesity, characterized by a body mass index (BMI) ≥ 30 kg/m^2^, is a global health concern, with its prevalence steadily increasing over the past few decades. It is a major risk factor for various metabolic disorders, cardiovascular diseases, certain cancers such as colorectal, renal, postmenopausal breast, and prostate cancers, and leukemia [1,4]. Understanding the mechanisms underlying obesity-related diseases is an area of intense research. Obesity is characterized by excessive adipose tissue accumulation and disrupts metabolic and endocrine functions [5]. This includes alterations in adipokine secretion, resulting in a chronic inflammatory state similar to that of the tumor microenvironment, which promotes tumor growth [1].

The tumor microenvironment is composed of cytotoxic immune cells, antigen-presenting cells (macrophages), and other cells such as fibroblasts, adipocytes, and extracellular matrix (ECM), which create an inflammatory state similar to that of damaged tissues [6]. Tumor cells modify the regulatory system of non-cancerous cells, leading to the invasion of pro-inflammatory factors such as cytokines, growth factors, and immune cells. This, in turn, triggers responses to damage, including the activation of the coagulation cascade and platelet activation factors. Consequently, endothelial dysfunction and fibrosis occur [1].

Metabolic reprogramming takes place within the tumor microenvironment. The “Warburg effect” is a phenomenon in which tumor cells prefer glycolysis to oxidative phosphorylation for ATP production, even under aerobic conditions. Despite lower ATP production and reduced energy efficiency, this metabolic preference aids cell division and metastasis [7].

This review aims to provide an in-depth analysis of the role of adipokines in health and disease, with a particular focus on their impact on tumor development and progression. Considering the increasing incidence of obesity-associated cancers, understanding the interplay between adipokines and tumorigenesis holds great promise for the identification of potential therapeutic targets and novel cancer prevention and treatment approaches.

## 2. Adipokine

### 2.1. Adipokine: Concept and Functions

Adipose tissue contains one of the most diverse cell types and includes adipocytes, endothelial cells, mast cells, fibroblasts, immune cells, and stem cells. Originally regarded as an inert energy reservoir, adipose tissue is now recognized as a vital endocrine organ that actively participates in the regulation of various physiological processes through adipokine secretion [1,4].

Adipokines are a class of bioactive molecules predominantly secreted by adipocytes and are the primary cellular constituents of adipose tissue [8]. Adiponectin, leptin, resistin, and visfatin have been the most extensively studied among various adipokines. Adipokines are vital mediators of complex interactions between adipose tissue and other organs [9]. Adipokines have diverse functions and encompass various biological activities, including immune responses, inflammation, glucose metabolism, insulin sensitivity, cell adhesion, angiogenesis, and appetite and satiety regulations at the whole-body level [10] (Table 1).

### 2.2. Roles of Key Adipokines

#### 2.2.1. Leptin

Leptin is a 16 kDa protein that plays a central role in regulating energy homeostasis and body weight [11]. It is primarily produced and secreted by adipocytes and its concentration is proportional to body fat mass [12]. Leptin serves as a crucial signal to the brain through hypothalamic receptors and provides information regarding the body’s energy reserves. In response to elevated leptin levels, appetite is suppressed and energy expenditure is increased, thus contributing to maintaining body fat mass within a narrow range [13].

In obesity, leptin levels are elevated and act as pro-inflammatory adipokines [14], inducing certain cytokines such as tumor necrosis factor-alpha (TNF-α), interleukin (IL)-1, and IL-6 [15,16]. Moreover, leptin contributes to the maintenance of insulin resistance, which is associated with obesity-associated diseases [11]. The dysregulation of leptin signaling contributes to the pathogenesis of obesity and metabolic disorders.

The leptin receptor belongs to the class I cytokine receptor family and has six splice variants. The leptin transmembrane receptor (Ob-R) isoforms (ObRa-ObRf) are divided into three classes based on their differences in length: long (ObRb), short (ObRa, ObRc, ObRd, and ObRf), and secretory (ObRe) isoforms [17]. Ob-R has an intracellular domain of sufficient length to initiate various signaling pathways that contribute to tumor cell survival, growth, and metastasis [18]. Leptin interacts with different receptor isoforms and activates various cellular pathways, resulting in diverse biological effects.

The short isoforms participate in leptin transport and clearance. ObRa and ObRc traverse the blood–brain barrier, whereas ObRe, derived from the external portion of ObRb, primarily acts as a protein that transports leptin in the bloodstream, regulating the serum leptin concentration by inhibiting the surface binding and endocytosis of leptin [19,20]. ObRb, with a long intracellular domain, has the ability to transmit intracellular signals and is mainly expressed in immune cells and throughout the central nervous system (CNS), particularly in the hypothalamus, contributing to the role of leptin in energy balance [20].

**Figure 1:**
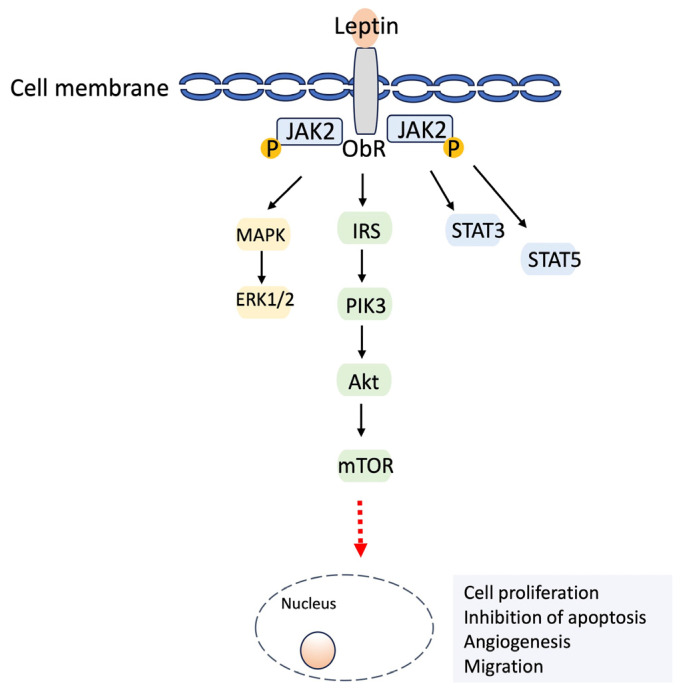
A schematic illustration of leptin-induced signaling pathways. The binding of leptin to its receptor (ObR) leads to the formation of the ObR/JAK2 complex, which results in phosphorylation (P). This phosphorylation activates MAPK/ERK1/2 signaling, PI3K/Akt, and downstream signals such as mTOR. Also, phosphorylated STAT3 and STAT5 translocated to the nucleus activate target genes. This leptin-induced signaling pathway promotes cell proliferation, inhibits apoptosis, and facilitates angiogenesis and migration.

When leptin binds to its receptor, it forms the ObR/JAK2p complex, activating phosphatidylinositol 3-kinase (PI3K), mitogen-activated protein kinase (MAPK), and signal transducer and activator of transcription (STAT) signaling pathways. Activation of the mammalian target of rapamycin (mTOR), the target of PI3K/protein kinase B (Akt), plays a crucial role in cell growth and survival [12,21]. High Ob-R expression has been observed in breast and gastrointestinal cancers, indicating an unfavorable prognosis [14,21]. A schematic of the leptin-induced signaling pathways is summarized in Figure 1.

#### 2.2.2. Adiponectin

Adiponectin is the most abundant adipokine secreted mainly from visceral adipose tissue [12]. Adiponectin exists in various forms such as homomultimers, trimers, hexamers, and high molecular weight (HMW) multimers in cells and plasma [22] and combines with various receptors to exhibit distinct functions [23]. The ratio of HMW to low-molecular-weight oligomers is associated with insulin sensitivity. As a result, the effect of adiponectin differs according to the relative circulating concentration, form, and expression of its receptor subtypes (ADIPOR1 and ADIPOR2) [24]. Upon binding with its receptor, adiponectin triggers the activation of a number of downstream signaling pathways. The effects of adiponectin are primarily conveyed through the AMP-activated protein kinase (AMPK) and peroxisome proliferator-activated receptor (PPAR) pathways. Activating AMPK leads to the activation of sirtuin 1 (SIRT1), which plays a crucial role in adiponectin’s regulation of glucose and lipid homeostasis. Additionally, it enhances the activity of endothelial nitric oxide synthase (eNOS), facilitating the interaction between adiponectin and endothelial cells.

Furthermore, AMPK activation suppresses PI3K and mTOR signaling, resulting in a cytoprotective effect of adiponectin. Through suppressors of cytokine signaling 3 (SOCS3), adiponectin inhibits the activation of STAT3, which promotes the proliferation, survival, and invasion of cancer cells [17,25]. The pleiotropic functions of adiponectin include maintaining energy homeostasis; exerting anti-inflammatory actions such as inhibiting macrophages, T lymphocytes, and natural killer cells; promoting anti-apoptosis; and facilitating pro-angiogenic activities [26,27].

Adiponectin has various beneficial effects on metabolism and vascular health, including insulin-sensitizing and anti-inflammatory effects. Adiponectin exerts insulin-sensitizing effects, enhancing glucose uptake and utilization in peripheral tissues while inhibiting hepatic glucose production, leading to improved insulin sensitivity and glycemic control [17]. 

In addition to its metabolic roles, adiponectin exerts anti-inflammatory effects and modulates vascular function [4]. It suppresses the production of pro-inflammatory cytokines, such as TNF-α and IL-6, thereby mitigating chronic low-grade inflammation associated with obesity. Relevant to tumor development, adiponectin may exert indirect effects by enhancing cell sensitivity to insulin or through anti-inflammatory actions [12].

In contrast to other adipokines, adiponectin levels are inversely associated with adipose tissue mass. Higher adiponectin concentrations are observed in lean individuals, whereas obesity reduces adiponectin secretion [12].

#### 2.2.3. Visfatin

Visfatin, a protein with a molecular weight of 52 kDa, is synthesized in various tissues of the body. It was initially identified as a pre-B cell colony-enhancing factor that contributes to the growth and development of pre-B cells. Notably, in females characterized by visceral obesity, visfatin levels are often elevated [28]. Several studies have indicated that visfatin levels tend to increase with BMI and decrease with weight loss [29]. The secretion of circulating visfatin primarily occurs in visceral fat tissue, where macrophages are believed to play a more significant role in visfatin release than adipocytes. Visfatin has been suggested to function as a pro-inflammatory substance [30,31] and exhibits insulin-like effects and anti-apoptotic activity that impede neutrophil apoptosis [30,31,32].

#### 2.2.4. Resistin

Resistin is a 10-kDa polypeptide predominantly secreted by adipocytes. Secreted resistin circulates as homotrimers, homohexamers, and higher molecular weight oligomers [4]. It induces the production of pro-inflammatory cytokines, including TNF-α and IL-12 [33,34]. Resistin, referred to as ‘found in inflammatory zone 3′, was first discovered in 2001 [35] and is known to be linked to insulin resistance and the development of type 2 diabetes mellitus, especially in obese individuals [36].

Resistin triggers a proinflammatory state. Resistin enhances the inflammatory processes in adipose tissues, with adipocytes serving as target cells. Resistin can induce the production of IL-6, IL-8, and TNF-α in adipose tissue [37]. The properties of resistin, such as its ability to induce the expression of matrix metalloproteinases (MMPs) and vascular endothelial growth factor receptor (VEGFR) as well as the formation of endothelial cell tubes, may be associated with its malignancy [38].

#### 2.2.5. Apelin

Apelin, also known as APJ endogenous ligand, functions as an endogenous ligand for the G-protein-coupled apelin receptor (APJ), which was initially isolated from bovine stomach extract [39]. Synthesized as an immature single peptide of a 77-amino acid preprotein, apelin exists in various forms, including apelin-12, apelin-13, apelin-17, and apelin-36 identified as the most biologically active variants.

Apelin plays a pivotal role in diverse physiological processes, including apoptosis, inflammation, and tumor proliferation, while simultaneously contributing to angiogenesis and cell migration [40]. In cancer development, apelin is implicated in activating the apelin receptor, APJ, which is overexpressed in tumor tissues [41]. The apelin and APJ receptor systems have been suggested to regulate autophagy, apoptosis, and angiogenesis, thereby influencing tumor development [42].

#### 2.2.6. Chemerin

Chemerin, also known as retinoic acid receptor responder 2 (RARRES2), is abundant in adipose tissue and the liver. Human chemerin is a 163 amino acid protein chemically activated by C-terminal processing, with chemerin 157 being the most active. It mediates different cellular responses through two receptors: chemokine-like receptor 1 (CMKLR1) and G protein-coupled receptor 1 (GPR1) [43,44].

Considered an immunoregulatory protein, chemerin can act as either an anti-inflammatory or a pro-inflammatory mediator depending on the context [45]. In obese individuals, circulating chemerin levels increase, potentially contributing to dyslipidemia, low-grade inflammation, hypertension, and insulin resistance [46].

Similar to its role in inflammation, where chemerin can act as both a pro- and anti-inflammatory factor, its impact on cancer can be anti-tumor or cancer-promoting in the specific disease context [45].

#### 2.2.7. Omentin

Omentin-1, also known as intelectin-1, is released by adipocytes in visceral adipose tissue and its gene is located in chromosomal region 1q22–q23 [47]. With a molecular weight of approximately 35 kDa, omentin-1 comprises 313 amino acids. Interestingly, its concentration was negatively correlated with BMI [48].

Levels of omentin-1, whether low or high, could potentially serve as indicators of cancer progression, with some data suggesting an association between increased omentin levels in blood serum and cancer development [49]. Conversely, other data propose a potential decrease in omentin concentration with the malignancy of cancer [50]. However, the role of omentin in cancer development remains controversial and warrants further investigation.

### 2.3. Adipokine Secretion and Regulation in the Organism

Adipose tissue serves as the primary source of adipokines and the extent of adipose tissue mass, composition, and distribution significantly affects the overall adipokine secretion profile. In particular, changes in adipokine secretion within adipose tissue in obesity appear to contribute to obesity-related conditions [9].

#### 2.3.1. Adipose Tissue Depots

Excessive adipose tissue can arise from an increased number of adipocytes (hyperplasia) or an increased size of individual adipocytes (hypertrophy) [51]. Among these, hypertrophy has been identified as a crucial factor affecting insulin sensitivity and the secretion of pro-inflammatory adipokines [52,53,54,55]. In contrast to adipocyte hyperplasia, which typically maintains normal function, hypertrophic adipocytes tend to secrete higher levels of pro-inflammatory factors, such as leptin, IL-6, and monocyte chemoattractant protein-1 (MCP-1), while reducing the secretion of insulin-sensitizing factors such as adiponectin and IL-10 [56,57]. 

Furthermore, the impaired expandability of the subcutaneous adipose tissue, which results in abnormal ectopic fat deposition, can contribute to inflammation and adipose tissue dysfunction. Different fat deposits exhibit distinct adipokine secretion patterns. The subcutaneous adipose tissue beneath the skin tends to release higher levels of beneficial adipokines, including adiponectin. Conversely, visceral adipose tissues around internal organs are associated with increased secretion of pro-inflammatory adipokines, such as TNF-α and IL-6.

The subcutaneous adipose tissue has a high capacity for vascularization; however, this capacity decreases as fat mass accumulates. As subcutaneous adipose tissue fails to expand proportionally to caloric excess, fat is abnormally stored in ectopic depots, such as the omentum, mesentery, liver, and other organs [3,58,59,60,61]. Visceral obesity has been found to be more strongly linked to insulin resistance and metabolic disorders than to peripheral or subcutaneous obesity [62,63,64,65]. Relative changes in the proportions of these diverse fat deposits influence the overall adipokine environment, subsequently affecting metabolic and inflammatory responses.

#### 2.3.2. Obesity and Adipokine Dysregulation

Obesity is a prominent driver of adipokine dysregulation and exerts profound and far-reaching effects on the secretion and function of these bioactive molecules. This complex interplay is primarily fueled by the excessive accumulation of energy, which manifests as an increase in body weight and is primarily achieved through a combination of adipocyte hypertrophy (enlargement) and hyperplasia (increase in cell numbers). Adipocyte hypertrophy induces adipose tissue dysfunction. This dysfunctional state is characterized by several key features including decreased insulin sensitivity, hypoxia, increased intracellular stress, and tissue inflammation [3].

As adipose tissue undergoes expansion in response to obesity, a cascade of events occurs involving adipocytes and infiltrating immune cells, particularly macrophages. These dynamic interactions result in a significant shift in the secreted adipokine profile. This is characterized by loss of insulin sensitivity, increased secretion of pro-inflammatory adipokines, such as leptin, TNF-α, IL-6, and IL-1β, and reduced levels of beneficial adipokines like adiponectin [3]. These pro-inflammatory molecules contribute to the establishment of a chronic low-grade inflammatory state within the adipose tissue, perpetuating the detrimental effects of obesity.

In addition, obesity-associated adipocyte hypertrophy and hypoxia can contribute to the secretion of chemokines such as MCP-1, which further attracts immune cells to the adipose tissue and perpetuates a state of chronic low-grade inflammation. In summary, obesity-induced alterations in adipokine secretion and associated inflammatory state play pivotal roles in the pathogenesis of metabolic disorders and tumorigenesis [66].

## 3. Adipokines and Their Connection with Tumorigenesis

### 3.1. Association between Obesity and Cancer 

Obesity, characterized by the excessive accumulation of adipose tissue, is a major risk factor for various types of cancers. Epidemiological studies have consistently demonstrated positive associations between obesity and the incidence and mortality of several cancers, including endometrial, breast, liver, colorectal, and prostate cancers [21]. 

The underlying mechanisms connecting obesity to cancer development are complex and multifactorial and involve alterations in adipokine secretion, chronic inflammation, insulin resistance, and sex hormone perturbations [67,68,69]. 

As BMI increases, circulating insulin levels tend to increase, leading to the development of insulin resistance in obese individuals. Obesity-related insulin resistance is a well-known risk factor for the development of breast cancer, particularly in postmenopausal women [70]. Hyperinsulinemia can increase the risk of cancer via two mechanisms. The increase in insulin levels can act as a direct growth-promoting signal and the elevated free bioactive insulin-like growth factor 1 (IGF1) can indirectly alter the cellular environment favorably for tumor development. In addition, the activation of insulin and IGF1 receptors can trigger cancer-relevant intracellular signaling cascades that are essential for mitogenesis, anti-apoptosis, and angiogenesis [71,72,73]. The chronic inflammatory condition of adipose tissue in obesity shares similarities with the tumor microenvironment, which may contribute to tumor growth [1,74].

However, certain reports highlight the existence of the “obesity paradox”, indicating that a higher BMI before treatment may result in a poorer prognosis but post-treatment, overweight, and early obese states are associated with enhanced cancer survival. Obesity may function as an energy reserve and positively affect the metabolism of therapeutic drugs. William et al. demonstrated an increased anti-tumor effect in obese patients undergoing checkpoint blockade immunotherapy [66]. The paradoxical effect of obesity on cancer outcomes could be influenced by the pro-inflammatory effect of leptin, potentially enhancing the efficacy of immune checkpoint inhibitors [70]. It is crucial to recognize that this obesity paradox is not a universal characteristic found in all cancers and interpreting obesity as having a “protective” effect in cancer patients should be approached with caution, considering the potential involvement of various biases. However, additional data are required to better understand this phenomenon [75].

### 3.2. Potential Mechanisms of Adipokines in Tumorigenesis 

Adipokines, bioactive molecules secreted by adipose tissue, play a pivotal role in unraveling the intricate connection between obesity and cancer. In the tumor microenvironment, non-cancer cells such as adipocytes and macrophages interact to amplify inflammation and disrupt the balance of adipocytokine production. Consequently, these events culminate in a cascade of consequences, including the reprogramming of cancer cell metabolism, facilitation of tumor invasion and metastasis, and disruption of immune clearance mechanisms [76].

According to recent studies, many obesity-related genes have intronic sequences containing microRNA (miRNA) genes, which are susceptible to modulation by adipokines in peripheral tissues. Adipokines can exert either oncogenic or antitumor effects, depending on the function of the target gene regulated by adipokine-regulated miRNAs [4,77]. 

Several essential prerequisites are required to enable the advancement of tumor cells. These include the inhibition of apoptosis and the promotion of angiogenesis to ensure adequate oxygen and nutrient supply. Metastatic cancer cells also undergo metabolic aerobic glycolysis (Warburg effect) and acquire mesenchymal characteristics during metastasis [14].

Leptin and adiponectin are the most extensively studied adipokines in obesity-related cancers and there has been increasing interest in recent years toward new adipokines such as resistin and visfatin (Table 2).

#### 3.2.1. Leptin

Leptin is produced in proportion to the fat mass; therefore, its expression is elevated in obesity. Increased leptin and Ob-R expressions have been observed in various malignant tissues, including breast, lung, colon, uterine, liver, and ovarian cancers [78]. Several experimental studies have shown that, despite obesity, models in which the expression of leptin or leptin receptors was inhibited did not exhibit an increased risk of mammary cancer [79,80,81], suggesting that leptin signaling plays a crucial role in tumorigenesis rather than weight gain itself. Leptin has been implicated in diverse processes such as inflammation, inhibition of apoptosis, immune suppression, and angiogenesis, both by itself and in synergy with vascular endothelial growth factor [12,14,82]. Leptin triggers several pathways related to cell survival and proliferation. These include the Janus kinase (JAK)/signal transducer, STAT, PI3K/Akt, and mitogen-activated extracellular signal-regulated kinase/extracellular signal-regulated kinase ½ pathways [83].

High leptin levels are associated with an increased risk of esophageal adenocarcinoma in obese individuals. Leptin, secreted by peritumoral adipocytes, may be linked to cancer progression in esophageal adenocarcinomas. In esophageal squamous cell carcinoma, leptin expression correlates with lymph node involvement and tumor stage [84]. In gastric cancer, Ob-R expression is associated with poor prognosis, particularly in poorly differentiated gastric cancer [85]. Strong leptin expression is associated with poor prognosis. Leptin increases the expression of matrix metalloproteinases, which degrade ECM components, thereby affecting gastric cancer invasion [86]. In colorectal cancer, high leptin levels are associated with lymph node involvement, microvascular invasion, and advanced tumor stage [87]. Leptin is believed to exert a pro-migratory effect by activating the STAT and JAK signaling pathways, thereby influencing tumor progression. In addition, Ob-R mRNA expression was significantly higher in advanced colorectal cancer compared to in early stage disease [88]. 

Among gynecologic cancers, ovarian cancer is the deadliest disease. Similar to colorectal cancer, high Ob-R expression has been observed in the advanced stages of ovarian cancer compared to the early stages [89]. Leptin has been shown to promote cell migration and invasion in various in vitro studies on ovarian cancer. Leptin and Ob-R concentrations are positively correlated with cancer invasiveness, metastasis, poor prognosis, and aggressive cancer presentation [90].

An increase in BMI is associated with a higher incidence of breast cancer. Elevated leptin expression in breast cancer is indicative of a more aggressive cancer phenotype and is associated with tumor size, lymph node involvement, and metastasis [91]. Katarzyna et al. found that post-treatment serum leptin concentration may be associated with the prognosis of early stage luminal invasive breast cancer and can be used as a prognostic indicator [92]. The influence of leptin on tumor-associated macrophages (TAMs) is intriguing. TAMs are considered to exhibit a pro-tumor effect, behaving as M2 macrophages and promoting the epithelial–mesenchymal transition (EMT). Leptin stimulates the M2 macrophages, thereby promoting breast cancer progression. Although a decrease in adiponectin expression has been observed in breast cancer, the exact role of adiponectin in female cancer remains unclear and requires further research [14].

#### 3.2.2. Visfatin

Visfatin is an adipokine that regulates tumorigenesis. Several studies have provided compelling evidence for the influence of visfatin on various facets of cancer development, including cancer cell growth, angiogenesis, migration, and invasion [93,94]. The mechanisms underlying its oncogenic effects involve the PI3K/Akt, MAPK, and STAT [31,95]. Increased visfatin expression has been observed in tumor tissues of breast, pancreatic, and kidney cancers compared to adjacent non-cancerous tissues [95]. Moreover, elevated visfatin levels have been associated with unfavorable prognoses in breast, gastric, liver, and urothelial cancers [96]. 

#### 3.2.3. Resistin

Plasma resistin levels are elevated in female patients with breast cancer and high levels are associated with the highest histological grade, independent of age, body mass index, serum glucose, or menopause [97]. Furthermore, elevated resistin levels have been observed in lymphomas [98]. Resistin can induce angiogenesis, promoting functions such as VEGFR expression and the formation of endothelial cell tubes [38]. 

#### 3.2.4. Other Adipokines

Other adipokines including apelin and chemerin, which are overexpressed in obesity, exhibit oncogenic properties [99]. Hu et al. revealed a correlation between apelin expression and various clinicopathological parameters including tumor size, stage, histological type, lymph node metastasis, and adverse prognosis in breast cancer. The identification of apelin as a potential prognostic factor suggests its utility as a novel therapeutic target in breast cancer [100]. Gourgue et al. suggested that apelin may be a major factor contributing to tumor growth and metastasis in triple-negative breast cancer in obese patients [101]. Lin, Z. et al. described that chemerin, along with CA153, can be used as a biomarker for breast cancer diagnosis, as the serum levels of chemerin in breast cancer patients were higher than those in healthy groups [102]. Chemerin is a pleiotropic protein that has a tumor-promoting effect; however, its role in cancer is controversial as it can also exert antitumor effects by recruiting innate immune defenses and initiating growth-inhibitory downstream signaling [45]. However, the precise underlying mechanism remains unclear. 

#### 3.2.5. Adiponectin

In contrast, adiponectin exerts tumor-suppressive effects by inhibiting cell proliferation and inducing apoptosis by suppressing the cell regulatory cycle [17]. Adiponectin is one of the few adipokines whose production is downregulated during obesity and has anti-inflammatory actions that may interfere with the functions of immune cells, such as macrophages, T lymphocytes, and NK cells [26]. The antitumor effects of adiponectin are mediated through the membrane receptors ADIPOR1 and ADIPOR2, which activate signaling pathways related to apoptosis induction and cell cycle arrest. In addition, it downregulates survival and proliferative signals, such as MAPK, JAK/STAT, mammalian target of rapamycin, and Wnt/β-catenin [103,104]. Epidemiological evidence consistently demonstrates an inverse relationship between circulating adiponectin levels and the risk of obesity-related cancers, including endometrial, breast, advanced prostate, colorectal, and renal cancers [103,105]. 

Several studies have proposed that adiponectin, even without tumor formation initiation, may also contribute to tumor progression [17]. For example, it has been shown to exhibit proangiogenic actions in mouse models of breast tumors [106,107]. Some reports have shown that low adiponectin levels in obese individuals can induce the growth and migration of estrogen receptor (ER)α-positive cells and the invasion of breast cancer cells [108,109,110,111,112]. Mauro et al. proposed that the observed divergent effects of adiponectin on breast cancer growth may be attributed to the distinct modulation of cyclin D1 (CD1) levels mediated by ERα expression [113]. Therefore, adiponectin may have multifaceted roles. However, whether adiponectin has cancer-protective or cancer-promoting effects remains controversial [21].

#### 3.2.6. Omentin-1

Omentin-1, a pro-apoptotic and anti-inflammatory adipokine, undergoes changes in concentration in liver, colorectal, prostate, gastric, and breast cancers [47,114,115]. In addition, lower omentin-1 levels have been linked to an increased incidence of endometrial cancer and postmenopausal breast cancer [115,116]. Tahmasebpour et al. reported a significant downregulation in the gene expression of omentin-1 in breast cancer tissues compared to normal tissues. Furthermore, they observed elevated serum levels of omentin-1 in patients with p53-positive breast cancer compared with those in patients with p53-negative breast cancer. Consequently, these findings suggest that omentin-1 has a potential prognostic value for breast cancer [117].

The dysregulation of adipokine signaling can disrupt the delicate balance between pro- and anti-tumorigenic signals, creating a tumor-promoting microenvironment. Moreover, adipokines can influence the tumor immune response and alter the tumor–stroma interaction, further influencing tumor progression. Investigating the specific molecular pathways through which adipokines affect tumorigenesis is critical for developing targeted therapeutic interventions.

### 3.3. Impact of Adipokines on the Tumor Microenvironment (TME) and Metastasis 

The TME is a complex network of various components that surround the tumor, encompassing a diverse array of cellular and noncellular elements. These components include immune cells, fibroblasts, endothelial cells, adipocytes, blood vessels, and noncellular ECM components. Within this dynamic milieu, these elements engage in constant and intricate interactions with the tumor, influencing each other and ultimately driving tumor growth and progression [118]. 

**Figure 2:**
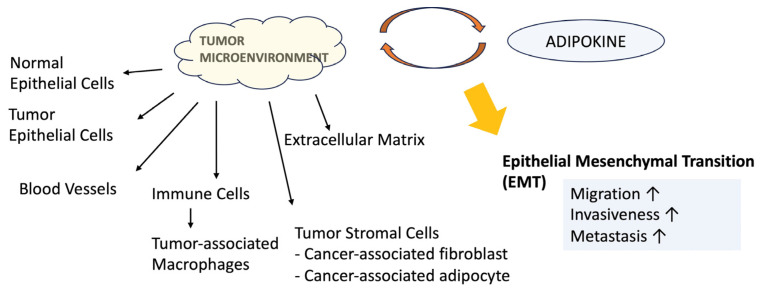
An illustrative schematic describes the diverse components of the tumor microenvironment, consisting of cellular and non-cellular parts. The interaction between adipokines and the tumor microenvironment disrupts normal extracellular matrix (ECM) and endothelial cell functions. As a result, these interactions activate the epithelial–mesenchymal transition (EMT), which is crucial for tumor malignancy and metastasis. High leptin and visfatin levels but low adiponectin levels promote these actions.

Adipokines interact with the TME, often resulting in the upregulation of various inflammatory cytokines, recruitment of immune cells (such as macrophages and lymphocytes), and disruption of normal ECM and endothelial cell functions [119]. Consequently, these interactions contribute to the activation of EMT, a crucial process in tumor malignancy and metastasis. A schematic representation of these processes is shown in Figure 2.

EMT is a cellular process by which endothelial cells lose their characteristic polarity and cell adhesion, thereby acquiring the morphological and functional features of mesenchymal cells. This results in increased migration, proliferation, resistance to apoptosis, and an enhanced ability to generate ECM components. One of the defining characteristics of EMT is a reduction in E-cadherin expression. EMT is associated with alterations in the intracellular cytoskeleton and extracellular matrix (ECM) degradation, leading to local invasion and subsequent dissemination into distant tissues [120]. 

Through their paracrine and endocrine effects, adipokines influence immune cell infiltration, polarization, and activation within the tumor microenvironment. This can lead to the establishment of an immunosuppressive milieu, favoring tumor immune escape. In addition, adipokines regulate tumor-associated fibroblasts and endothelial cells, thereby influencing tumor angiogenesis and tissue remodeling. 

Leptin has the potential to affect the tumor microenvironment through various mechanisms, resulting in tumor invasion and distant metastasis. It influences the pro-inflammatory, angiogenic, and fibrotic factors within the TME. Leptin is involved in the expression of matrix metalloproteinases, the activation of the transforming growth factor-β signaling pathway, and the engagement in the process of the epithelial–mesenchymal transition, all of which are crucial for cancer cell migration [14]. High levels of leptin were observed in the TME blood.

Furthermore, TAMs are the principal immune cells within tumors and play distinct roles depending on their polarization state. M1 macrophages are associated with pathogen clearance and antitumor effects, whereas M2 macrophages exhibit protumorigenic effects and contribute to tumor cell invasion and progression [121]. Differentiation and polarization of these macrophages are regulated by various signals and cytokines present in the TME. Leptin activates M2 macrophages and promotes the production of IL-6, IL-8, IL-12, IL-18, and TNF-α [122,123]. Upregulation of IL-18 by leptin facilitates the migration and progression of breast cancer cells and is also implicated in metastasis in gastric cancer, melanoma, and tumor growth in colorectal cancer [17].

Leptin is involved in the loss of epithelial cell characteristics, promoting the expression of mesenchymal features and leading to the migration and invasion of tumor cells. This unfavorable impact on prognosis has been observed in several types of cancers. Emerging evidence has highlighted the potential risks associated with persistent leptin exposure in normal breast tissue. This exposure can induce EMT, a transformative process that increases susceptibility to breast cancer development [124]. Furthermore, the influence of leptin-induced EMT is not confined to breast cancer; it has also been well documented in various other cancer types, including esophageal adenocarcinoma, cholangiocarcinoma, lung cancer, and prostate cancer. In these contexts, the role of leptin in driving EMT contributes significantly to the enhancement of cell invasion and migration, ultimately underscoring the profound effect of leptin on the metastatic potential of tumor cells [17].

Adiponectin is the most abundant adipokine in the TME. However, its precise role has not yet been fully elucidated. There is an inverse correlation between circulating adiponectin levels and several tumor antioxidant markers.

Adiponectin also serves as a crucial regulator of macrophage proliferation and differentiation. When adiponectin is deficient, tumor growth is thought to increase due to a decrease in the recruitment of macrophages to tumor cells. Adiponectin promotes M2 macrophage expression.

Adiponectin inhibits tumor growth in melanoma, lung cancer, and rhabdomyosarcoma [17]. In contrast to leptin, adiponectin is involved in the reversal and inhibition of the EMT, thereby playing a role in preventing cancer progression. The insulin-like growth factor-I receptor (IGF-IR) is important for maintaining EMT and adiponectin exerts an antagonistic effect on IGF-IR signaling [125].

As mentioned above, the dysregulation of adipokine can create a microenvironment that promotes tumor growth and may impact tumor progression. Investigating the influence of adipokines on tumor development and understanding the molecular pathways are critical for future interventions in cancer treatment.

## 4. Conclusions

Adipokines play critical roles in the complex relationship between obesity and tumorigenesis. Their multifaceted effects on various facets of cancer development, including tumor growth, metastasis, and the tumor microenvironment, highlight their potential as valuable therapeutic targets and biomarkers for cancer prevention and treatment. Understanding the interplay between adipokines and cancer provides a foundation for the development of personalized approaches to combat obesity-related cancers and improve patient prognosis.

Our review aimed to comprehensively outline the impact of various adipokines on tumor development and provide insights for potential therapeutic applications in the future.

However, most studies adopted a cross-sectional approach, neglecting the exploration of temporal changes in individual adipokines. Moreover, the diversity in cancer types, methodologies, and the limited number of studies pose challenges in drawing detailed conclusions specific to each cancer type. Caution is advised when interpreting the results owing to the prevalent moderate or high risk of bias attributed to the study design and reporting shortcomings.

## Figures and Tables

**Table 1:** The biological activities of adipokines in health [9].

Adipokine	Main Actions
Apelin	Inhibits insulin secretion
Chemerin	Chemoattractant protein; regulates adipogenesis
Leptin	Regulates appetite, food intake, energy expenditure, fertility, and other processes
Lipocalin 2	Related to insulin resistance and inflammation
MCP-1	Inflammation of adipose tissue
Omentin	Anti-inflammatory; insulin sensitizing

**Table 2:** Summary of actions of adipokines depends on the cancer type.

Cancer Type	Adipokine	Actions
Esophageal adenocarcinoma	Leptin ↑	Correlates with lymph node involvement and tumor stage
	Adiponectin ↓	Low expressions compared to healthy control group
Gastric cancer	Leptin ↑	Ob-R expression is associated with poor prognoses and increases the expression of matrix metalloproteinases, which degrade ECM components
	Adiponectin ↓	Low expression is associated with an increased risk for gastric cancer
Colorectal cancer	Leptin ↑	Lymph node involvement, microvascular invasion, and advanced tumor stage
	Resistin ↑	Overexpression in a human colon cancer cell line
	Adiponectin ↓	Suppression of colon cancer cells by its receptor-mediated AMPK activity
Gynecologic cancer	Leptin ↑	Promote cell migration and invasion in various in vitro studies on ovarian cancer
	Adiponectin ↓	Inversely associated with endometrial cancer
Breast cancer	Leptin ↑	Associated with tumor size, lymph node involvement, and metastasis
	Visfatin ↑	Associated with poor prognoses
	Resistin ↑	Elevated expressions in breast cancer
	Apelin ↑	Parameters including tumor size, stage, histological type, lymph node metastasis, and adverse prognoses
	Chemerin ↑	Elevated expressions in breast cancer
	Omentin-1 ↓	Low level in postmenopausal breast cancer
